# Alterations in glutathione redox homeostasis among adolescents with obesity and anemia

**DOI:** 10.1038/s41598-021-82579-5

**Published:** 2021-02-04

**Authors:** Dalal Alkazemi, Abdur Rahman, Banan Habra

**Affiliations:** Department of Food Science and Nutrition, College of Life Sciences, Kuwait University, AlShadadiyah, Kuwait

**Keywords:** Biomarkers, Diseases

## Abstract

The reduced (GSH)-to-oxidized (GSSG) glutathione ratio represents a dynamic balance between oxidants and antioxidants. However, redox status in adolescents with obesity and anemia has not been investigated. This study investigated the association of erythrocyte GSH redox status (GSH, GSH:GSSG ratio, and glutathione peroxidase [GPx] activity) with anemia and adiposity in adolescents. This case–control study nested in a cross-sectional study enrolled 524 adolescents (268 boys; 256 girls). The prevalence of anemia in overweight and obesity (OWOB) was 5.2% in boys and 11.7% in girls. The GSH:GSSG ratio and GPx activity were significantly higher in girls than in boys (p < 0.001), in anemic than in non-anemic subjects (p < 0.001), and in OWOB than in normal-weight subjects (p < 0.001). Similarly, significantly higher GSH: GSSG level (p < 0.001) and GPx activity (p < 0.001) were found in subjects with 90th percentile waist circumference than in those with < 90th percentile. GPx and GSH:GSSG were positively associated with anemia after adjusting for age, sex, and body mass index (adjusted odds ratio, adjOR [95% confidence interval, CI] 2.18 [1.44–3.29]) or tertiles (adjOR [95% CI], T3 = 2.49 [1.03–6.01]). A similar association was noted for GSH and GPx. A compensatory increased redox defense mechanism exists in anemia and obesity among adolescents without metabolic disturbances.

## Introduction

Obesity and iron deficiency anemia are two common global childhood disorders that have a significant impact on lifelong health. Accumulating evidence indicates that children and adolescents with obesity are at a greater risk of iron deficiency (ID) and iron deficiency anemia (IDA) than their normal-weight (NW) peers^1,2^. Although the relationship between obesity and anemia has not been fully delineated, obese subjects appear to be at a greater risk of IDA due to several risk factors such as chronically low iron intake and increased blood volume due to a higher adipose tissue mass^3^. Obesity is a state of chronic low-grade inflammation associated with increased circulatory levels of proinflammatory cytokines that have been proposed to link anemia and obesity^3,4^. High levels of interleukin (IL)-1, IL-6, and tumor necrosis factor (TNF)-α in obesity have been indicated to induce the dysregulation of the iron regulatory hormones hepcidin and lipocalin-2. This dysregulation leads to low circulatory iron and intracellular iron overload in adipose tissues^5–8^. The generation of reactive oxygen species (ROS) in obesity appears to be triggered by the increased levels of proinflammatory cytokines and by the intracellular iron overload, which then dysregulates adipocytes’ mitochondrial function^7^. This phenomenon creates a vicious cycle in which increased levels of adipokines generate ROS, and high levels of ROS dysregulate body weight by modulating satiety and appetite signals at the hypothalamic level^9–11^. Thus, the dysregulated iron homeostasis is not only the consequence of obesity but could also be involved in the pathogenesis of obesity-related morbidities such as insulin resistance and diabetes^7^.

An imbalance in the antioxidant network through the enhanced generation of ROS or depletion of antioxidants leads to oxidative stress. Sulfhydryl-rich molecules play a critical role in the antioxidant network. Glutathione (GSH), a tripeptide, is a key component in its reduced form in the cellular antioxidant defense system^12,13^. Through a reaction catalyzed by a selenium-dependent enzyme, glutathione peroxidase (GPx), GSH donates its hydrogen to the highly reactive ROS and converts them into more stable compounds. In such reactions, two GSH molecules dimerize through a disulfide bond to form oxidized glutathione (GSSG)^14^. The ratio of GSH to GSSG (GSH:GSSG ratio) is a commonly used sensitive early biomarker of the whole-body redox status^15,16^. Cellular alterations in the GSH:GSSG ratio are fundamental in the fine-tuning of signal transduction, including oxidative stress signaling needed for physiological events such as cell cycle regulation, and other cellular processes^16^. GPx is an important modulator of the balance between GSH and GSSG; therefore, its measurement provides a comprehensive view of the redox status along with the GSH:GSSG ratio.

Although oxidative stress in obese pediatric populations has been well demonstrated, there has been discordance in terms of redox biomarkers. Higher erythrocyte GPx activity and GSH levels were noted in children with obesity than in their NW counterparts, in contrast to increased oxidative stress indicators in obese children, such as oxidized low-density lipoprotein, malondialdehyde, and advanced oxidation products^17–21^. Thus far, the relationship between ID states and antioxidant enzymes, including GPx, has been inconsistent and controversial^22,23^. The studies report variation ranging from an increase in the antioxidant enzyme activity in IDA^24^ to a decrease^23^ or no effect^25–28^. The redox status of adolescents with obesity and anemia has not been investigated. Such studies would provide important evidence regarding the role of oxidative stress in the progression of metabolic dysregulation and the subsequent development of obesity and anemia-related comorbidities with their transition into young adulthood.

Kuwait has one of the highest rates of obesity in the adult and pediatric populations^29^. Childhood and adolescent obesity in Kuwait is particularly alarming, as up to 45% of children in the age group 5–19 years have either overweight or obesity^30–32^. Although anemia is not a major public health problem in Kuwait, it is still a public health issue in children and adolescents. The Kuwait Nutritional Surveillance System has consistently reported a 15–20% prevalence of anemia among school-age children^32^. A more recent study conducted on a nationally representative sample of adolescents reported the prevalence of anemia as 8.1% (11% in girls and 5% in boys)^33^. These reports provide preliminary support for the coexistence of both obesity and anemia in adolescents in Kuwait.

In this study, the prevalence of anemia in adolescents with overweight and obesity (OWOB) in Kuwait was assessed, and the relationship of erythrocyte GSH redox status (GSH, GSH:GSSG ratio, and GPx activity) with anemia and obesity was examined based on (a) measures of obesity in terms of body mass index (BMI) and waist circumference (WC) and (b) IDA status.

## Results

### Prevalence of obesity and anemia

The sociodemographic characteristics of the subjects (N = 524; 268 boys and 256 girls) are summarized in Table 1. The median (IQR) BMI percentile was 80.90 (48.93–95.88); 15.3% (10.8% boys, 19.9% girls; p = 0.006) of subjects were overweight, and 27.9% (26.9% boys, 28.9% girls) were classified as obese. When OWOB groups were combined, the total prevalence of excess adiposity was 43.1% in the overall sample (37.7% in boys and 48.8% in girls, p < 0.05). Based on the WC percentile obesogenic cutoff, 15.5% (17.9% boys and 12.9% girls; p = 0.073) were above the cutoff point.

**Table 1:** Sociodemographic characteristics of the study subjects (N = 524).

Age (years), mean (SD)	12.61 (0.62)	N (%)
Sex	Boys	269 (51.2)
Nationality	Kuwaiti	405 (77.1)
	Non-Kuwaiti Arab	117 (22.3)
	Non-Kuwaiti non-Arab	3 (0.6)
Father’s education level (Data missing for 13 cases)	Up to intermediate education	101 (19.7)
	Secondary school	124 (24.2)
	Diploma	99 (19.3)
	University or higher	188 (36.7)
Mother’s education level (Data missing for 9 cases)	Up to intermediate education	75 (14.5)
	Secondary school	120 (23.3)
	Diploma	114 (22.1)
	University or higher	207 (40.1)
Father’s income (Data missing for 16 cases)	1000 KD or less	136 (26.7)
	1001–2000 KD	238 (45.3)
	More than 2000 KD	71 (13.9)
	Do not know	64 (12.6)
Mother’s income (Data missing for 96 cases)	1000 KD or less	226 (52.7)
	1001–2000 KD	137 (27.3)
	More than 2000 KD	14 (3.3)
	Do not know	72 (16.8)
School governorate	Asimah	68 (13)
	Hawally	64 (12.2)
	Farwaniya	108 (20.6)
	Jahra	117 (22.3)
	Mubarak Al-Kabeer	80 (15.2)
	Ahmadi	88 (16.8)

The prevalence of anemia was 13% (8.2% in boys and 18% in girls; p = 0.001) (Table 2). The prevalence of anemia was 19.5% in subjects with OWOB and 8.1% in subjects with NW (p < 0.05). In boys, the prevalence of anemia in the OWOB group was 13.9% compared with 4.8% in the NW group (p < 0.05). In girls, the prevalence of anemia in the OWOB group was 24.0% compared with that of 12.2% in the NW group (p < 0.05). In the non-anemic overall sample, the prevalence of overweight was 39.9%, whereas it was 64.7% in anemic children (p < 0.05). A similar pattern was observed for boys and for girls separately. In boys, 59.3% were non-anemic and NW, 32.5% were non-anemic and OWOB, 3.0% were anemic and NW, and 5.2% were anemic and OWOB (Table 3). Among girls, 44.9% were non-anemic and NW, 37.1% were non-anemic and OWOB, 6.3% were anemic and NW, and 11.7% were anemic and OWOB. Significant interactions between boys and girls in terms of both weight and anemia statuses were observed. In the overall sample, the prevalence of anemia was 2.4 times higher in the OWOB group than in the NW group (p < 0.05). When stratified by sex, the prevalence of anemia in the OWOB group was 2.9 times higher in boys and 2.0-fold higher in girls than that in the NW group (p < 0.05 for both sexes). With regard to the weight status of anemic and non-anemic subjects, the prevalence of OWOB was 1.6-fold higher in anemic subjects than in non-anemic subjects (p < 0.05). When stratified by sex, the prevalence of OWOB in anemic subjects was higher by 1.8-fold in boys and by 1.4-fold in girls than that in non-anemic subjects (p < 0.05 for both sexes). The proportion of girls in the OWOB and anemic group was more than twice that of boys (Table 3).

**Table 2:** Coexistence of anemia and overweight/obesity in adolescents.

	Non-anemic	Anemic	Total	% Anemia
**Total**				
Normal weight	274	24	298	8.1
Overweight and obesity	182	44	226	19.5
Total	456	68	524	13.0
% Overweight and obese	39.9	64.7	43.1	
**Boys**				
Normal weight	159	8	167	4.8
Overweight and obesity	87	14	101	13.9
Total	246	22	268	8.2
% Overweight and obese	35.4	63.6	37.7	
**Girls**				
Normal weight	115	16	131	12.2
Overweight and obesity	95	30	125	24.0
Total	210	46	256	18.0
% Overweight and obese	45.2	65.2	48.8

**Table 3:** Classification of subjects (%) with anemia and weight status.

	Boys	Girls
Normal-weight and non-anemic (NW–NA)	59.3	44.9
Overweight/obese and non-anemic (OWOB–NA)	32.5	37.1
Normal-weight and anemic (NW–A)	3.0	6.3
Overweight/obese and anemic (OWOA–A)	5.2	11.7

### Redox status in relation with sex, anemia, and adiposity

The erythrocyte concentrations of GSH, GSH:GSSG ratio, and GPx activity varied by sex, anemia status, and weight status, based on both BMI and WC categories (Table 4). GSH levels, in µmol/g Hb, (mean [standard deviation, SD]) were higher in girls than in boys (2.47 [0.67] vs. 1.69 [0.68], p < 0.001); in anemic subjects than in non-anemic subjects (2.60 [0.80] vs. 1.99 [0.74], p < 0.001); and higher in subjects with OWOB than in those with NW (2.31 [0.68] vs. 1.93 [0.80], p < 0.001). Similarly, the GSH:GSSG ratio was higher in girls than in boys (1.06 [0.48] vs. 1.00 [0.47], p < 0.001); in anemic subjects than in non-anemic subjects (1.10 [0.55] vs. 1.02 [0.46], p < 0.001); and in subjects with overweight/obesity than in those with NW (1.05 [0.44] vs. 1.01 [0.24], p < 0.001). The GPx activity, in U/g Hb, (median [IQR]) was higher in girls than in boys (95.16 [45.04–130.32] vs. 52.35 [39.53–104.20], p < 0.001); in anemic subjects than in non-anemic subjects (121.81 [92.86–154.60] vs. 57.83 [40.30–115.11], p < 0.001); and in subjects with OWOB than in those with NW (114.07 [62.46–137.69] vs. 48.11 [37.72–88.91], p < 0.001). Similarly, with WC percentile categories, those with ≥ 90th percentile had significantly higher levels of GSH:GSSG and GPx activity than those with < 90th percentile (GSH:GSSG, 1.08 [0.49] vs. 1.01 [0.48], p < 0.001; GPx, 98.32 [51.48–140.72] vs. 63.14 [41.32–118.44], p < 0.001), but the levels of GSH between the two waist categories were not different from each other.

**Table 4:** Levels of GPx, GSH, and GSH:GSSG.

	N	GPx (U/g Hb) Median (IQR)	GSH (µmol/g Hb) Mean (SD)	GSH:GSSG Mean (SD)
Total	524	70.33 (42.70–120.48)	2.07 (0.78)	1.30 (0.23)
**Sex**				
Boys	268	52.35 (39.53–104.20)	1.69 (0.68)	1.00 (0.47)
Girls	256	95.16 (45.04–130.32)	2.47 (0.67)	1.06 (0.48)
p-value		< 0.001	< 0.001	< 0.001
**Age**				
Gr 1, 12 years	243	62.04 (40.33–112.91)	2.06 (0.77)	1.03 (0.5)
Gr 2, 13 years	244	82.48 (43.80–126.22)	2.10 (0.81)	1.03 (0.47)
Gr 3, 14 years	37	57.27 (41.66–110.11)	1.94 (0.61)	1.01 (0.41)
p-value		0.182	0.460	0.652
**Anemia status**				
Anemic	68	121.81 (92.86–154.60)	2.60 (0.80)	1.10 (0.55)
Non-anemic	456	57.83 (40.30–115.11)	1.99 (0.74)	1.02 (0.46)
p-value		< 0.001	< 0.001	< 0.001
**BMI**				
Normal weight	298	48.11 (37.72–88.91)^a^	1.93 (0.80)^a^	1.01 (0.24)^a^
Overweight	80	114.07 (62.46–137.69)^b^	2.31 (0.68)^b^	1.05 (0.44)^b^
Obese	146	106.78 (56.77–133.79)^b^	2.22 (0.72)^b^	1.07 (0.47)^b^
p-value		< 0.001	< 0.001	< 0.001
**WC percentile adjusted for sex and age**				
Non-obesogenic waist	442	63.14 (41.32–118.44)	2.04 (0.76)	1.01 (0.48)
Obesogenic waist	81	98.32 (51.48–140.72)	2.20 (0.83)	1.08 (0.49)
p-value		0.002	> 0.05	< 0.001
**WC:Ht ratio tertiles**				
T1: < 0.45	131	48.34 (37.45–103.57)^a^	1.79 (0.71)^a^	0.99 (0.44)^a^
T2: ≥ 0.45–< 0.56	264	64.86 (42.50–18.44)^a^	2.14 (0.78)^b^	1.03 (0.48)^b^
T3: ≥ 0.56	128	101.22 (53.61–142.13)^b^	2.07 (0.77)^b^	1.08 (0.48)^c^
p-value		< 0.001	< 0.001	< 0.001

In the categories of sex, anemia status, and WC percentile, the GPx data were analyzed using the chi-square test, and the GSH and GSH:GSSG data were analyzed using the independent-samples t-test. In the categories of age, BMI, and WC:Ht tertiles, GPx data were analyzed using the independent-samples median test, whereas the GSH and GSH:GSSG ratio data were analyzed using one-way ANOVA. p-values are for two-tailed tests. The GSH:GSSG ratio was transformed into square root, and mean (SD) values were converted back into the original form by squaring them. Superscript letters denoting values in the same column in the same category indicate significant differences.

*GPx* glutathione peroxidase, *Hb* hemoglobin, *IQR* interquartile range, *GSH* reduced glutathione, *SD* standard deviation, *GSSG* glutathione disulfide, *BMI* body mass index, *WC* waist circumference, *Ht* height.

### Sex-wise comparisons between the combined anemia and weight categories

**Figure 1:**
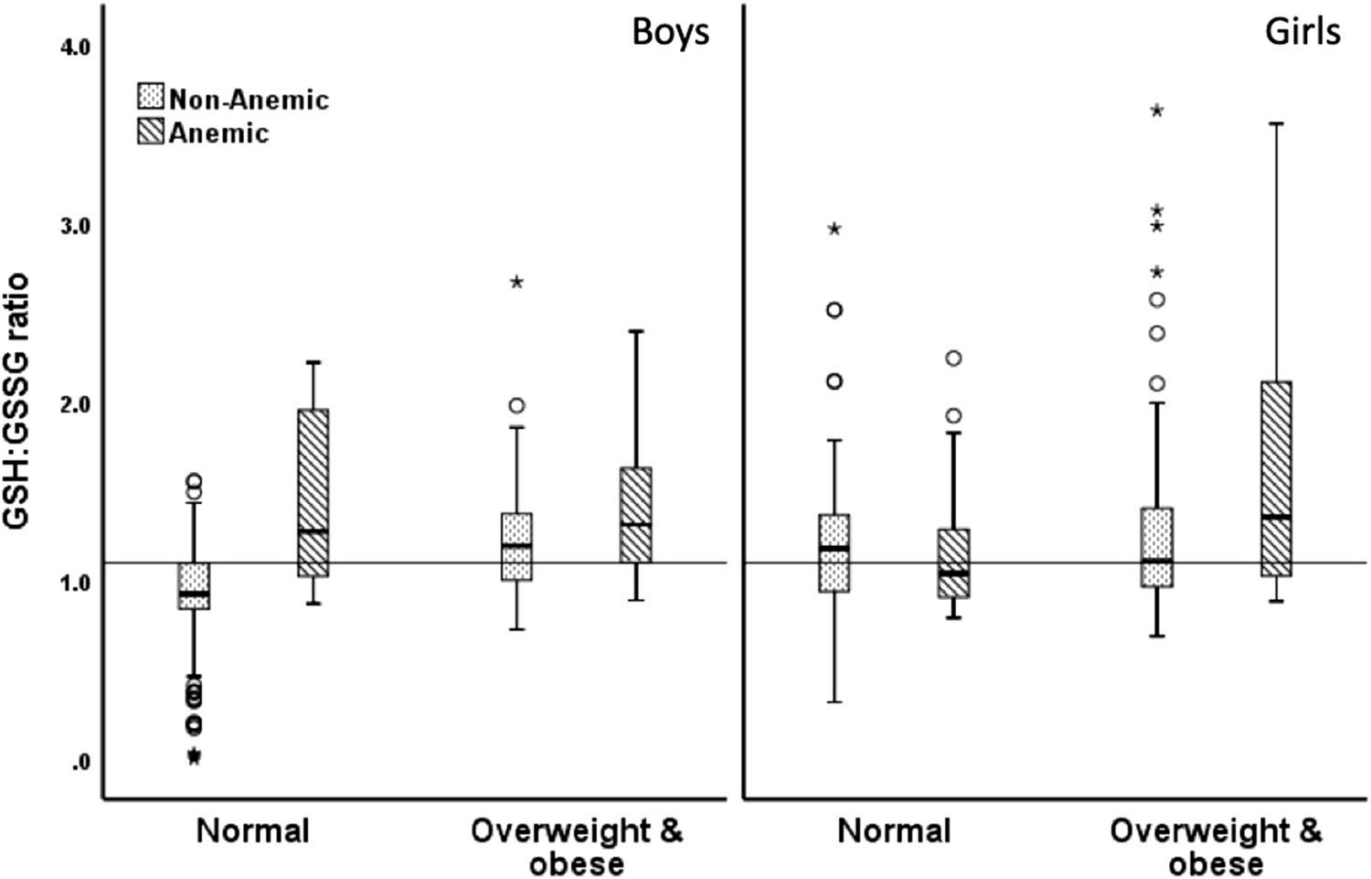
Reduced glutathione-to-glutathione disulfide ratio (GSH:GSSG ratio) in relation to the anemia status.

**Figure 2:**
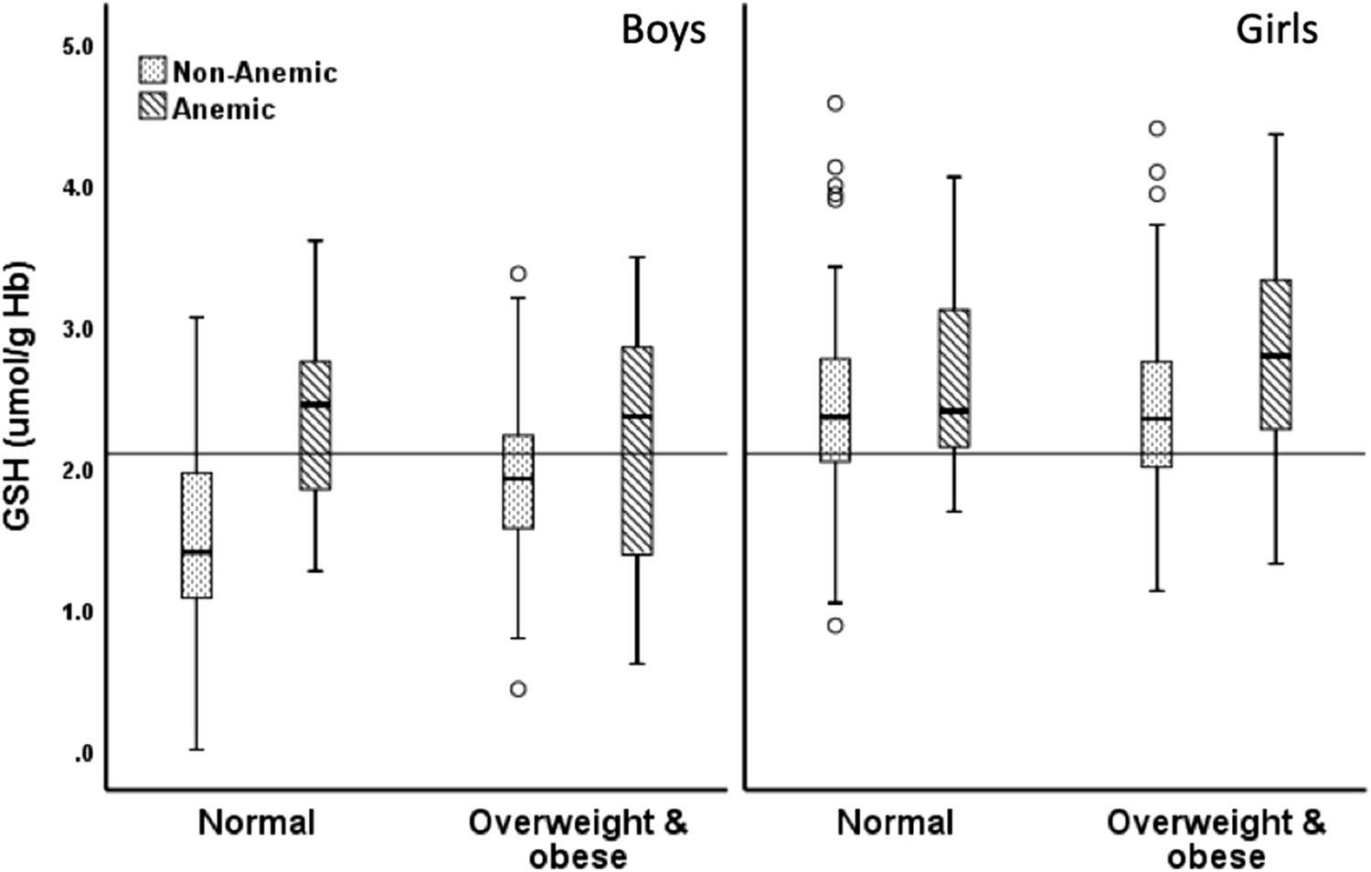
Reduced glutathione (GSH) levels in relation to the anemia status.

**Figure 3:**
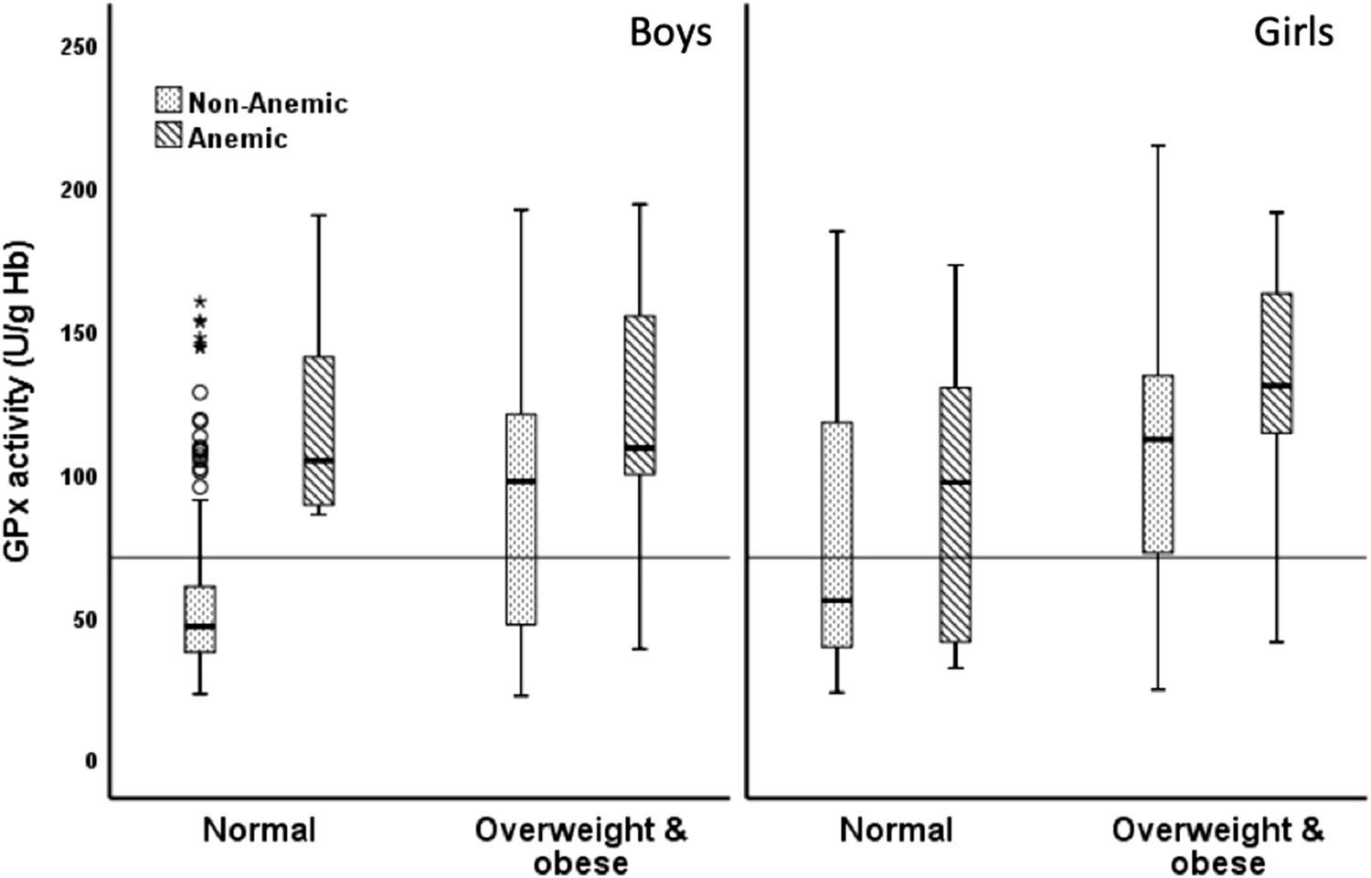
Glutathione peroxidase (GPx) activity in relation to the anemia status. The horizontal line indicates the overall median.

For the combined category of weight and anemia statuses in each sex (Fig. 1), anemic overweight boys, anemic NW boys, and non-anemic overweight boys had a significantly higher GSH:GSSG ratio than non-anemic NW boys (1.31 [1.06–1.70], 1.27 [0.89–2.23], and 1.19 [0.99–1.38] vs. 0.92 [0.84–1.10], respectively; p < 0.05). These observations were also similar in terms of GSH concentrations in µmol/g Hb, (Fig. 2; 2.36 [1.35–2.87], 2.45 [1.32–2.89], and 1.92 [1.56–2.24], vs. 1.40 [1.08–1.97], respectively; p < 0.05) and GPx activity, in U/g Hb, (Fig. 3; 108.58 [98.13–155.51], 104.24 [88.60–157.93], and 97.05 [46.72–121.11], vs. 46.25 [37.20–60.43], respectively; p < 0.05). Among girls, the differences in the median GSH:GSSG ratio were not significant among the four groups (Fig. 1; 1.35 [1.23–2.16], 1.04 [0.90–1.31], 1.11 [0.97–1.42], and 1.18 [0.94–1.37]). Anemic girls who were overweight had the highest level of GSH when compared with non-anemic overweight girls (2.79 [2.26–3.35] vs. 2.35 [1.99–2.75], p = 0.02) and non-anemic NW girls (2.79 [2.26–3.35] vs. 2.36 [2.03–2.77], p = 0.018; Fig. 2). Similarly, anemic overweight girls had the highest level of GPx activity when compared to non-anemic overweight girls (130.21 [113.13–164.19] vs. 111.49 [68.38–135.03], p = 0.036), anemic NW girls (130.21 [113.13–164.19] vs. 96.45 [40.18–133.79], p = 0.03), and non-anemic NW girls (130.21 [113.13–164.19] vs. 55.39 [39.10–144.26], p < 0.001) (Fig. 3).

### Association of redox status with anemia and obesity in adolescents

The Spearman’s correlation analysis (Table 5) showed that GSH:GSSG ratio, GSH, and GPx activity were positively associated with anemic status (ρ = 0.181, p < 0.001; ρ = 0.251, p < 0.001; and ρ = 0.215, p < 0.001, respectively). Similarly, GSH:GSSG ratio, GSH, and GPx activity were negatively associated with Hb concentration (ρ = − 0.224, p < 0.001; ρ = − 0.301, p < 0.001; and ρ = − 0.149, p < 0.001, respectively). In terms of measures of adiposity, the GSH:GSSG ratio was positively correlated with BMI (ρ = 0.255, p < 0.001), WC (ρ = 0.317, p < 0.001), and WC:Ht (ρ = 0.333, p < 0.001). GSH and GPx activity also showed similar associations (Table 5). Sex was also positively correlated with GSH:GSSG ratio, GSH, and GPx activity, whereas age was significantly negatively correlated only with GSH: GSSG (ρ = − 0.111, p < 0.05). In multinomial logistic regression (Table 6), the GSH:GSSG ratio was positively associated with anemia status after adjusting for age, sex, and BMI categories, regardless of whether it was used as a continuous variable (adjusted odds ratio, adjOR [95% CI], 2.18 [1.44–3.29]; p < 0.001, R2 = 0.138) or as tertiles (adjOR [95% CI], T3 = 2.49 [1.03–6.01], T2 = 1.54 [0.67–3.56]; p < 0.001, R2 = 0.104). A similar association was observed for GSH (R2 = 0.154 as continuous and R2 = 0.126 as tertiles) and GPx (R2 = 0.179 as continuous and R2 = 0.139 as tertiles). When increased adiposity, as BMI categories, was examined in the multinomial logistic regression model adjusted for age, sex, and anemia status; GSH:GSSG ratio, GSH, and GPx activity remained significantly positively associated with similar magnitudes (Table 7).

**Table 5:** Spearman’s correlation coefficient (ρ) for the correlation between redox status and measures of adiposity and anemia.

	GPx	GSH	GSH:GSSG
Age	0.059	− 0.036	− 0.111**
Sex	0.215*	0.506*	0.212*
Anemia	0.278*	0.251*	0.181*
Hb (g/dL)	− 0.149*	− 0.301*	− 0.224*
BMI	0.343*	0.225*	0.255*
WC	0.264*	0.197*	0.317*
WC:Ht ratio	0.274*	0.204*	0.333*

Age, sex, and anemia were used as categorical variables, while BMI, WC, WC:Ht ratio, and Hb were used as continuous variables for assessing associations.

*GPx* glutathione peroxidase, *Hb* hemoglobin, *GSH* reduced glutathione, *GSSG* glutathione disulfide, *BMI* body mass index, *WC* waist circumference, *Ht* height.

*p < 0.01, **p < 0.05, (2-tailed).

**Table 6:** Association between anemia and redox status.

	OR [95% CI] Unadjusted	p-value	R^2^	OR [95% CI] Adjusted	p-value	R^2^
GSH:GSSG (continuous)	2.68 [1.76–4.09]	< 0.001	0.087	2.18 [1.44–3.29]	< 0.001	0.138
**GSH:GSSG tertiles**						
T1 < 1.14	Ref	0.002	0.045	Ref	< 0.001	0.104
T2 ≥ 1.14–< 1.60	2.11 [0.94–4.73]			1.54 [0.67–3.56]		
T3 ≥ 1.60	3.97 [1.73–9.12]			2.49 [1.03–6.01]		
GSH (continuous)	2.76 [1.94–3.94]	< 0.001	0.121	2.45 [1.64–3.66]	< 0.001	0.154
**GSH (tertiles)**						
T1 < 1.43	Ref	< 0.001	0.077	Ref	< 0.001	0.126
T2 ≥ 1.43–< 2.55	2.10 [0.89–4.96]			1.29 [0.51–3.24]		
T3 ≥ 2.55	5.64 [2.39–13.34]			3.26 [1.25–8.4]		
GPx (continuous)	1.02 [1.01–1.03]	< 0.001	0.156	1.02 [1.01–1.02]	< 0.001	0.179
**GPx tertiles**						
T1 < 42.70	Ref	< 0.001	0.102	Ref	< 0.001	0.139
T2 ≥ 42.70–< 120.53	1.88 [0.79–4.48]			1.65 [0.68–3.97]		
T3 ≥ 120.53	6.54 [2.78–15.38]			4.40 [1.78–10.85]		

Anemia was used as a binary variable (anemic vs. non-anemic); the non-anemic group was used as a reference in logistic regression.

The adjusted model is adjusted for age, sex, and BMI categories.

*GPx* glutathione peroxidase, *Hb* hemoglobin, *OR* odds ratio, *CI* confidence interval, *GSH* reduced glutathione, *SD* standard deviation, *GSSG* glutathione disulfide, *BMI* body mass index, *WC* waist circumference, *Ht* height.

**Table 7:** Association between obesity and redox status.

	OR [95% CI] Unadjusted	p-value	Nagelkerke Psuedo R^2^	OR [95% CI] Adjusted	p-value	Nagelkerke Psuedo R^2^
GSH:GSSG (continuous)	2.70 [1.76–4.140]	< 0.001	0.069	2.35 [1.50–3.67]	< 0.001	0.092
**GSH:GSSG tertiles**						
T1 < 1.14	Ref	< 0.001	0.122	Ref	< 0.001	0.150
T2 ≥ 1.14–< 1.60	3.726 [2.259–6.146]			3.84 [2.18–6.05]		
T3 ≥ 1.60	6.301 [3.593–11.050]			5.84 [3.27–10.44]		
GSH (continuous)	1.77 [1.40–2.26]	< 0.001	0.060	1.66 [1.26–2.20]	< 0.001	0.080
**GSH (tertiles)**						
T1 < 1.43	Ref	< 0.001	0.083	Ref	< 0.001	0.112
T2 ≥ 1.43–< 2.55	3.63 [2.25–5.87]			3.59 [2.15–5.99]		
T3 ≥ 2.55	3.31 [1.93–5.67]			2.90 [1.58–5.33]		
GPx (continuous)	1.02 [1.02–1.03]	< 0.001	0.217	1.02 [1.02–1.02]	< 0.001	0.224
**GPx tertiles**						
T1 42.70	Ref	< 0.001	0.142	Ref	< 0.001	0.157
T2 ≥ 42.70–< 120.53	2.55 [1.58–4.12]			2.49 [1.53–4.04]]		
T3 ≥ 120.53	7.48 [4.29–13.02]			6.50 [3.67–11.52]		

Weight status was used as a binary variable (normal-weight vs. overweight + obese).

The normal-weight group was used as reference in logistic regression.

The adjusted model is adjusted for age, sex, and anemia status categories.

*GPx* glutathione peroxidase, *Hb* hemoglobin, *OR* odds ratio, *CI* confidence interval, *GSH* reduced glutathione, *SD* standard deviation, *GSSG* glutathione disulfide, *BMI* body mass index, *WC* waist circumference, *Ht* height.

## Discussion

In the present study, erythrocyte GPx activity, GSH concentrations, and GSH:GSSG ratio were higher in adolescents with an OWOB status than in NW adolescents. Another novel finding was that the three redox erythrocyte parameters were also positively associated with anemia, even after adjusting for known covariates including sex, age, and obesity. In this regard, a significantly negative association was observed between Hb and redox status, including GSH and GPx activity. Of the three redox parameters studied, GPx activity showed the highest percent change in association with weight status and anemia. In the context of both obesity and anemia, it is possible that the observed increase in GPx antioxidant activity and the redox status was due to an appropriate adaptive response to the chronic oxidative stress that, as suggested previously, occurs in diabetic patients and smokers^34^. Although GPx provides protection against oxidative stress and modulates redox-mediated cellular signaling, excessive GPx activity exerts deleterious effects due to the reduction in essential cellular oxidants and the subsequent reductive stress^35^. Lack of essential cellular oxidants diminishes cell growth and dysregulates mitochondrial function and cellular metabolism. Excess GPx and reducing equivalents (GSH) have been linked to cardiomyopathy, cardiac dysfunction, and development of insulin resistance, a common feature of type 2 diabetes and metabolic syndrome^35^.

Although previous studies have not examined redox status in the adolescent obese population, Rowicka et al.^36^ and Kilic et al.^37^ reported a significantly higher total antioxidant capacity in children with obesity than in NW controls. Higher total oxidant capacity or oxidative stress index levels together with an elevated total antioxidant capacity were reported in children. Abuali^18^ reported increased activities of antioxidant enzymes (superoxide dismutase, GR, and GPx) in overweight children (mean age 9.7 ± 1.5 years); however, contrary to the findings of the present study, the above enzymatic parameters were decreased in the obese group in the previous study. A decrease in the GPx activity in obese children has been attributed to a depletion in the level of erythrocyte GSH, which is a cofactor for GPx^38,39^. The apparent contradictory results for obese adolescents described in the present work could indicate that the GSH status, particularly GPx, depends on the degree of adiposity and/or age of the subjects. Duration of adiposity, metabolic health status, degree of disease development and/or severity, and sex may also contribute to variations in the antioxidant status in response to obesity^19,40^. Further support for this notion comes from studies in which obese children with insulin resistance showed an impaired systemic oxidative status in contrast to obese children without insulin resistance or healthy controls. In these studies, obese children without insulin resistance, despite a transient decrease in GSH levels during the glucose tolerance test, were able to recover and maintain normal levels of lipid peroxides, similar to control subjects^41^. The ability of the antioxidant system to respond to an acute stressor in obese children without insulin resistance may be mediated through a more beneficial oxidative stress profile, with higher levels of principal antioxidants (GSH) together with lower levels of lipid peroxidation, possibly through shared anti-inflammatory pathways^8,42^.

The findings of the present study are consistent with those of some studies with regard to the redox status in anemia^24,43^, which indicates an increased activity of antioxidant defense enzymes in red blood cells during anemia. This can be an adaptive mechanism during the oxidative stress context of anemia, whereby sustained increased levels of erythrocyte H_2_O_2_ induce GPx and other antioxidant enzymes^24,43^, resulting in an increased redox status. However, considerable variations exist in human studies with regard to ID and redox status^44^, which can be attributed to the study differences in sample size, age of subjects, distribution of males and females in the sample, and severity of ID. For example, Altun et al.^24^ found no increase in GPx activity in groups of infants with IDA when compared with aged-matched controls. It was suggested that this discrepancy was due to the immaturity of the antioxidant capacity of infants, where it could not generate an adequate response to stress with GPx activity increase^24^.

In terms of sex-specific changes, while the values of the redox status (erythrocyte GSH concentrations, GPx activity, and the GSH:GSSG ratio) were higher in girls, the percent change associated with overweight and anemia was higher in boys. This discord was particularly evident when comparing the NW and overweight groups in the non-anemic category. This observation can be explained by the disproportionately higher percentage of girls in the anemic overweight groups than that of boys. GSH metabolism and GSH-dependent responses in humans appear to be regulated by both sex hormones, progesterone and testosterone^45^. In premenopausal women, the activity of erythrocyte GPx was found to be higher than that in healthy postmenopausal women. Furthermore, GPx activity in premenopausal women was significantly higher than that in age-matched men, but it was not significantly different between postmenopausal women and age-matched men^46^. Such findings suggest that there is a sex-dependent intensification of the antioxidant response, with girls being more resistant to the negative effects of an increased adiposity status than boys^47^.

OWOB in adolescents in Kuwait is a public health issue, and our result of > 40% of adolescents with OWOB is consistent with that of previous reports^48,49^. Kuwait and countries in the Arabian Gulf region have experienced a rapid transition from a traditional semi-urbanized life to a modern and urbanized society after the economic boom post the discoveries of oil since the 1960s. There has been a concomitant rise in the prevalence of obesity and other cardio-metabolic problems^50^. Adolescence is a critical period for the development and expression of obesity-related comorbidities in both sexes^51^. The prevalence of anemia in adolescents in the present study is in line with that mentioned in previous reports showing that anemia in adolescents is of mild public health significance, particularly in girls, as per the WHO classification (5.0–19.9%)^33,52^. There was, however, a disproportionately high prevalence of anemia in those with OWOB, which shows a clear coexistence of anemia and OWOB in this adolescent cohort. Similar to these findings, the coexistence of anemia and OWOB had been reported in many countries undergoing nutrition transition^53–55^. The combination of both anemia and obesity may affect adolescent growth and development, leading to psychological disorders, cognitive dysfunction, impaired motor abilities, altered timing of puberty, and multiple risk of comorbidities such as the metabolic syndrome and type 2 diabetes^55,56^.

The coexistence of both anemia and obesity was particularly high in girls, as the proportion of girls who were both overweight and anemic was more than twice that of boys. Increased iron loss in the menstrual blood and inadequate dietary iron intake have been suggested to contribute to a higher prevalence of anemia in girls^33,48^. The higher prevalence of both anemia and OWOB in adolescent girls, compared with that in boys, could be explainable by the higher consumption of energy-dense foods (sweets, cakes and donuts, burgers, French fries, and hot dogs), lower consumption of fruits and vegetables^48,49,57,58^, lower consumption of iron rich foods such as animal protein^59^, and lack of physical activity^48^. Therefore, adolescents with obesity may be at a higher risk of ID because of unbalanced meals, which are high in calories but low in iron, whether absolute or relative to body mass, together with increased iron needs.

To the best of our knowledge, this is the first study on the relationship between anemia and the redox status and GPx activity in a large sample of healthy adolescents. This study is among the few that put forward the concept of metabolic flexibility in overweight and obese adolescents and in relation to anemia through an increased antioxidant status as measured by the redox status and GPx activity. Most previous studies have been performed in infants, children, or adults and used biomarkers of lipid peroxidation or other biomarkers that are largely modulated by unknown factors. The present study had a powered sample size for the analyses and included both sexes equally. Another strength of this study is that it was based on a narrow age range, thus minimizing age-dependent variations. There are, however, a few limitations in this study. For example, not including inflammatory markers such as IL-1, IL-6, or C-reactive protein (CRP), markers of metabolic risk such as C-peptides or insulin resistance index, and oxidative stress markers such as plasma isoprostanes limited the interpretation of our findings to support the antioxidant compensation hypothesis, in particular in subjects with OWOB. In addition, the narrow age range of the study subjects that was included as a strength to minimize age-related variation limits the extrapolation of the results to other age groups such as children and adults. Furthermore, it has been reported that approximately 5–15% of the GSH could be oxidized to GSSG during sample deproteination with acids^60^. This could potentially result in the overestimation of GSSG and thus lower GSH:GSSG values. Derivatization of the GSH with N-ethylmaleimide has been suggested to overcome this analytical anomaly. However, this GSH-N-ethylmaleimide derivatization logistically was not possible for us as it is required during the sample collection, and we used sedimented erythrocytes provided to us from another study. The statistically significant increase in the GSH:GSSG ratio in cases vs controls, despite this apparent artifact if it has happened, suggests that the changes in the glutathione redox status are robust and could be detected despite this experimental anomaly. Finally, the cross-sectional nature of the study limits the interpretation of the findings to only correlations.

## Conclusions

In conclusion, adolescents with anemia and obesity with no other metabolic disturbances were shown to have increased erythrocyte GSH concentrations, GSH:GSSG ratio, and GPx activity. The increased glutathione redox status could be a compensatory mechanism in response to the increased oxidative burden due to anemia and obesity. Further studies are warranted to investigate the basis for the relationship between these redox parameters and other biomarkers of oxidative stress, and to investigate the mechanisms underlying the association of anemia and excessive adiposity with alterations in the redox status in adolescence. The effects, if any, of dietary modifications on the three redox measures need further investigation. The assessment of the redox status in relation to the progression of obesity-related comorbidities such as metabolic syndrome, diabetes, and cardiovascular disease (CVD) in cohort studies on adolescents could have important public health relevance.

## Methods

### Study design and participants

This present study was nested within a cross-sectional study conducted in public middle schools in Kuwait, as previously described^61,62^. In the original study, data were collected from 1426 children (701 boys) in the age range of 11–16 years, randomly selected from all the six Governorates of Kuwait, utilizing multi-stage random cluster sampling methods with a probability proportional to the size of each governorate. All students of public schools, both boys and girls, in grades 6, 7, and 8 (age range 11–14 years) were eligible for inclusion in the study. Children who had any systemic diseases (diabetes, cardiovascular, liver, or kidney diseases) or those who were on any medication for behavioral or psychological problems were excluded from the study. Data were collected from parents through a self-administered questionnaire and from study subjects through a face-to-face interview by trained data collectors. For this study, a nested case–control design was employed in which patients were selected based on their overweight/obesity status and control subjects were age- and sex-matched subjects, both recruited at a ratio of 1:1.6. The sample size was calculated based on the obesity prevalence of 20% in Kuwaiti adolescents for a case–control study, to detect a similar odds ratio with 90% statistical power and a 5% margin of error. The calculated sample size was 181 subjects per group. The final sample of 524 subjects included 291 with NW and 226 with overweight/obesity. Ethical approval was granted by the Ethics Committee of the Health Sciences Centre, Kuwait University (No. DR/EC/2338), and the Ethics Committee of the Ministry of Health, Kuwait (No. 2015/248 and No. 2017/1025 for additional tests). Written informed consent was obtained from the subjects’ parents, and verbal assent was obtained from all the study subjects before drawing blood. The study was conducted according to the ethical standards set by the Helsinki Declaration.

### Anthropometric measurements

Standing height (Ht) and body weight of the study subjects were measured in a standardized manner, using a digital weight and Ht scale (Detecto, Webb City, MO, USA) with the subjects standing erect without shoes and wearing light clothes. BMI-for-age z-scores (zBMI) were calculated using the World Health Organization (WHO) growth charts. A zBMI of ≥  + 3 SD was classified as obesity, while zBMIs of >  + 2 SD and <  + 3 SD were defined as overweight. Subjects with a zBMI of <  + 2 SD were classified as NW. We originally categorized zBMI into four levels: underweight, NW, overweight, and obese. We then found that < 1% of the subjects were underweight. Due to the small number of adolescents in this latter category, the underweight and NW categories were combined. WC was measured in the horizontal plane at the superior border of the right iliac crest to the nearest 0.1 cm with a non-stretchable tape by a trained data collector. Measurements were taken at the end of normal expiration; three readings were taken, and the average of the three was recorded. Care was taken to ensure that the tape was horizontal to the floor and touched the skin without compressing it. Population-based age- and sex-specific percentile cutoffs^63^ were used to categorize adolescent obesogenic waist (in cm) for boys aged 12, 13, and 14 years old and for girls 12, 13, and 14 years old. The WC cutoffs for boys were ≥ 91.5, ≥ 95.3, and ≥ 98.5, while the WC cutoffs for girls were ≥ 91.3, ≥ 95.2, and ≥ 98.5, respectively.

### Blood collection and biochemical analyses

Five milliliters of blood sample were collected by venipuncture by a trained nurse. One tube (EDTA-containing) was centrifuged at 16,000 × *g* for 10 min; plasma and buffy coats were separated, and the sedimented erythrocytes (1 mL) were collected in Eppendorf tubes and stored at − 80 °C for the redox status analysis. Classification of anemia was made according to hemoglobin (Hb) levels as described earlier^33^. Anemia was defined as Hb < 115 g/L in boys and girls aged younger than 12 years; Hb < 120 g/L in the 12–14 years age group; Hb < 130 g/L in boys aged 15 years or older; and Hb < 120 g/L in girls aged 15 years or older^64^.

### Determination of erythrocyte glutathione redox status

The erythrocyte GSH redox status was measured using the commercial GSH assay kit (Cat. #: 703002; Cayman Chemical, Ann Arbor, MI, USA) according to the manufacturer’s instructions. This kit utilizes GSH reductase for the quantification of GSH. Briefly, the erythrocyte samples stored at − 80 °C were thawed and lysed in four volumes of ice-cold high-performance liquid chromatography (HPLC)-grade water; samples were then deproteinized by adding an equal volume of metaphosphoric acid (5%) on ice for 10 min. The suspension was centrifuged at 16,000 × *g* for 10 min at 4 °C. The supernatant was collected and stored on ice, when used immediately, or frozen at − 80 °C for future use. The contents of total GSH (TGSH) and GSSG in the erythrocytes were determined; additionally, the level of reduced GSH (the difference between TGSH and GSSG) and the GSH:GSSG ratio were calculated.

### Assessment of erythrocyte GPx activity

GPx activity was determined using a kit assay (Cat. #: K762-100; BioVision, Milpitas, CA) as per the manufacturer’s instructions. In this assay, GPx reduced cumene hydroperoxide while oxidizing GSH to GSSG. The generated GSSG was then reduced to GSH with the consumption of NADPH by GR. The decrease in NADPH, which was measured colorimetrically at 340 nm, was proportional to GPx activity. The assay had a detection sensitivity of ~ 0.5 mU/mL of GPx in the samples.

### Statistical analysis

Data were coded, entered into, and analyzed using the Statistical Package for the Social Sciences (SPSS) for Windows version 24 (IBM Corp., Armonk, N.Y., USA). GSH was normally distributed, whereas GPx and the GSH:GSSG ratio were not normally distributed. GPx activity had a more bimodal distribution and could not be normalized with the usual transformation. The GSH:GSSG ratio was normalized by taking the square root of the variable. For normally distributed variables, data are presented as mean (SD), whereas for non-normally distributed variables, descriptive data are reported as the median and interquartile range (IQR). Significant group differences in the GPx activity were assessed using the chi-square test and the independent-samples median test, whereas for GSH and the GSH:GSSG ratio, the t-test for independent samples and analysis of variance (ANOVA) were used. The Spearman’s correlation coefficients (ρ) were calculated to determine the correlation between GSH, GSH:GSSG ratio, and GPx activity, with each of these variables treated separately as the dependent variable and WC, BMI, and Hb as the independent variables. Hb was used as a continuous variable, whereas the other variables were used as categorical ones. The association of GSH, GSH:GSSG ratio, and GPx activity with obesity or anemia was evaluated using multinomial logistic regression. The odds ratios were adjusted for age, sex, and anemia status (or obesity status). Anemia, BMI, and WC were used as binary categorical variables. The WC/Ht ratio was used as both a continuous variable and as tertiles. The analyses were conducted by fitting GSH, GSH:GSSG ratio, and GPX as continuous variables as well as categorizing them into tertiles. The level of significance was set at p < 0.05.

## Data Availability

Data are available from the corresponding author upon reasonable request.
